# AGAP1-associated endolysosomal trafficking abnormalities link gene–environment interactions in neurodevelopmental disorders

**DOI:** 10.1242/dmm.049838

**Published:** 2023-09-26

**Authors:** Sara A. Lewis, Somayeh Bakhtiari, Jacob Forstrom, Allan Bayat, Frédéric Bilan, Gwenaël Le Guyader, Ebba Alkhunaizi, Hilary Vernon, Sergio R. Padilla-Lopez, Michael C. Kruer

**Affiliations:** ^1^ Pediatric Movement Disorders Program, Barrow Neurological Institute, Phoenix Children's Hospital, Phoenix, AZ 85016, USA; ^2^ Departments of Child Health, Neurology, Genetics and Cellular & Molecular Medicine, University of Arizona College of Medicine Phoenix, Phoenix, AZ 85004, USA; ^3^ Institute for Regional Health Services, University of Southern Denmark, 5230 Odense, Denmark; ^4^ Department of Epilepsy Genetics and Personalized Medicine, Danish Epilepsy Center, 4293 Dianalund, Denmark; ^5^ Service de Génétique, CHU de Poitiers, 86000 Poitiers, France; ^6^ Laboratoire de Neurosciences Experimentales et Cliniques, INSERM U1084, 86000 Poitiers, France; ^7^ Department of Medical Genetics, North York General Hospital, Toronto, ON M3J0K2, Canada; ^8^ Division of Clinical and Metabolic Genetics, Department of Pediatrics, The Hospital for Sick Children, University of Toronto, Toronto, ON M3J0K2, Canada; ^9^ Department of Genetic Medicine, Johns Hopkins University, Baltimore, MD, USA; ^10^ Programs in Neuroscience, Molecular & Cellular Biology, and Biomedical Informatics, Arizona State University, Tempe, AZ 85287, USA

**Keywords:** *Drosophila*, AGAP1, Synaptic morphology, Endolysosome, Autophagy, eIF2α

## Abstract

*AGAP1* is an Arf1 GTPase-activating protein that regulates endolysosomal trafficking. Damaging variants have been linked to cerebral palsy and autism. We report three new cases in which individuals had microdeletion variants in *AGAP1*. The affected individuals had intellectual disability (3/3), autism (3/3), dystonia with axial hypotonia (1/3), abnormalities of brain maturation (1/3), growth impairment (2/3) and facial dysmorphism (2/3). We investigated mechanisms potentially underlying *AGAP1* variant-mediated neurodevelopmental impairments using the *Drosophila* ortholog *CenG1a*. We discovered reduced axon terminal size, increased neuronal endosome abundance and elevated autophagy compared to those in controls. Given potential incomplete penetrance, we assessed gene–environment interactions. We found basal elevation in the phosphorylation of the integrated stress-response protein eIF2α (or eIF2A) and inability to further increase eIF2α phosphorylation with subsequent cytotoxic stressors. *CenG1a*-mutant flies had increased lethality from exposure to environmental insults. We propose a model wherein disruption of *AGAP1* function impairs endolysosomal trafficking, chronically activating the integrated stress response and leaving AGAP1-deficient cells susceptible to a variety of second-hit cytotoxic stressors. This model may have broader applicability beyond *AGAP1* in instances where both genetic and environmental insults co-occur in individuals with neurodevelopmental disorders.

## Teaser

**Summary:** *AGAP1* deletion variants were identified in three individuals with neurodevelopmental disorders, and, using a loss-of-function *Drosophila* model, we show that *AGAP1* disruption impairs neuronal endolysosomal trafficking and chronically activates the stress response.

## INTRODUCTION

Known genetic contributions to neurodevelopmental disorders are rapidly expanding and include both Mendelian and complex (non-Mendelian) phenomena. Mendelian inheritance patterns include classic autosomal dominant, recessive and sex chromosome-linked monogenic disorders, currently estimated to lead to ∼20% of autism spectrum disorders (Mahjani et al., 2021) and ∼25% of cerebral palsy cases (Moreno-De-Luca et al., 2021; van Eyk et al., 2021). Autosomal dominant inheritance patterns represent important contributors to neurodevelopmental disorders, often arising spontaneously as *de novo* variants (Brunet et al., 2021), and may exhibit complex, non-Mendelian characteristics such as incomplete penetrance and variable expressivity (Ahluwalia et al., 2009). Other complex inheritance elements include epigenetic contributions to disease, gene–gene (epistatic) interactions and gene–environment interactions. Despite the inherent challenges in studying complex inheritance, such patterns represent important contributors to neurodevelopmental disorders such as cerebral palsy (Fahey et al., 2017). Although substantial advances have been made in statistical and causal modeling of complex genetic factors (Madsen et al., 2011), few experimentally validated genes and mechanisms have been described for complex inheritance leading to neurodevelopmental disorders such as cerebral palsy.

Missense variants in the Arf1 regulator gene *AGAP1* and heterozygous deletions in the 2q37.2 region that span this gene (Leroy et al., 2013) are associated with autism spectrum disorder (Cukier et al., 2014; Pacault et al., 2019) and cerebral palsy (Chopra et al., 2022; Jin et al., 2020; van Eyk et al., 2019). Despite a statistical enrichment in *AGAP1* variants in a cerebral palsy cohort (van Eyk et al., 2019), there is also evidence for environmental factors contributing to disease in several cerebral palsy patients with *AGAP1* variants. Therefore, the pathogenicity of these variants has not been definitively established and the disease–gene link for *AGAP1* is still unknown. Additionally, although the frequency is very low, not all individuals harboring *AGAP1* variants manifest neurodevelopmental disorders [Genome Aggregation Database (gnomAD), heterozygous loss of function (LoF)=0.008%; https://varsome.com/gene/hg38/agap1]. Such incomplete penetrance is particularly common in individuals with autism spectrum disorder (Geschwind, 2011), and many autism susceptibility genes have thus been referred to as ‘risk genes’ (Yuen et al., 2017). To better understand potential contributors to the apparent complex disease relationship of *AGAP1*, we sought to identify the underlying pathophysiology and characterize potential gene–environment interactions caused by *AGAP1* loss of function.

*AGAP1* is broadly expressed, with RNA levels highest in the brain and protein expression highest in the lung, endocrine tissues, male reproductive tissue, adipose tissue and bone marrow/lymphoid tissues (https://www.proteinatlas.org/ENSG00000157985-AGAP1/tissue). *AGAP1* is expressed in the mouse and human brain throughout pre- and post-natal development, with subcellular localization to dendrites, axons and synapses (Arnold et al., 2016). This suggests that AGAP1 plays a role in brain development, although studies of its function are limited. *AGAP1* is predicted to be intolerant to loss-of-function variants [observed/expected LoF=0.1169; LoF Z score=5.864; probability of loss-of-function intolerance (pLI)=1.0; https://varsome.com/gene/hg38/agap1] and potentially to missense variants as well (observed/expected missense ratio=0.8479; missense Z score=1.287; https://varsome.com/gene/hg38/agap1). AGAP1 contains a GTPase-like domain, Pleckstrin homology (PH) domain and GTPase-activating protein (GAP)-activity domain (http://www.ebi.ac.uk/interpro/protein/UniProt/Q9UPQ3/). The GAP activity of AGAP1 activates Arf1 hydrolysis to complete the GTPase cycle (Nie et al., 2003). The proper initiation and termination of Arf1 signaling is required for actin cytoskeleton polymerization (Davidson et al., 2015; Saila et al., 2020), which drives the formation of vesicles (Heuvingh et al., 2007). Overexpression of AGAP1 creates punctate endocytic structures and reduces stress fibers, demonstrating potential roles in regulating actin dynamics and trafficking from the endocytic compartment (Nie et al., 2003).

AGAP1 also regulates protein trafficking via the PH domain by recruiting subunits σ3 (AP3S3) and δ (AP3D1) of the AP-3 protein trafficking adaptor complex. AP-3 positively regulates the movement of proteins to the lysosome (Nie et al., 2003) and plasma membrane (Bendor et al., 2010). Immunostaining confirmed AGAP1 localization to early and recycling endosomes (Arnold et al., 2016) and to the Golgi (Cukierman et al., 1995). Both increases and decreases in AGAP1 levels decrease the rate of protein trafficking out of Rab11-positive endosomes (Arnold et al., 2016), and AGAP1-mediated endocytic recycling regulates the surface localization of M5 muscarinic receptor (CHRM5) and, consequently, neuronal function (Bendor et al., 2010). AP-3 is important for trafficking lysosome membrane proteins, such as LAMP1, from the Golgi to lysosomes (Chapuy et al., 2008). Therefore, AGAP1 is predicted to be a key regulator of protein trafficking from early endosomes to the Golgi, lysosomes or the plasma membrane.

To better understand how AGAP1 may contribute to nervous system function and expand on the potential *AGAP1* disease link, we report clinical phenotypes of three individuals with microdeletion variants that had not been previously reported and compare phenotypes with prior reports. We have previously described impaired locomotion from a loss-of-function variant of *CenG1a*, the *Drosophila* ortholog to *AGAP1* (Jin et al., 2020). *CenG1a* has well-conserved protein domains (Gündner et al., 2014) [*Drosophila* RNAi Screening Center Integrative Ortholog Prediction Tool (DIOPT), 13/16 tools support an orthologous gene-pair relationship; https://www.flyrnai.org/cgi-bin/DRSC_orthologs.pl] and *Drosophila* models have been widely used to dissect mechanisms of neurological disease (Ugur et al., 2016). Here, we investigated the role of *CenG1a* in neuronal morphology, endolysosomal distribution and composition, autophagy, and stress responses in this genetic model.

## RESULTS

### Patient clinical phenotypes

The dominant mutation assessment tool DOMINO (https://varsome.com/gene/hg38/agap1) predicts that heterozygous variants in *AGAP1* will lead to haploinsufficiency [probability of haploinsufficiency or P(HI)=0.8548]. *AGAP1* is also intolerant to genomic loss-of-function variants (pLI=0.9994). These observations provide complementary evidence for their role in *AGAP1*-associated neurodevelopmental disorders.

**Fig. 1.**
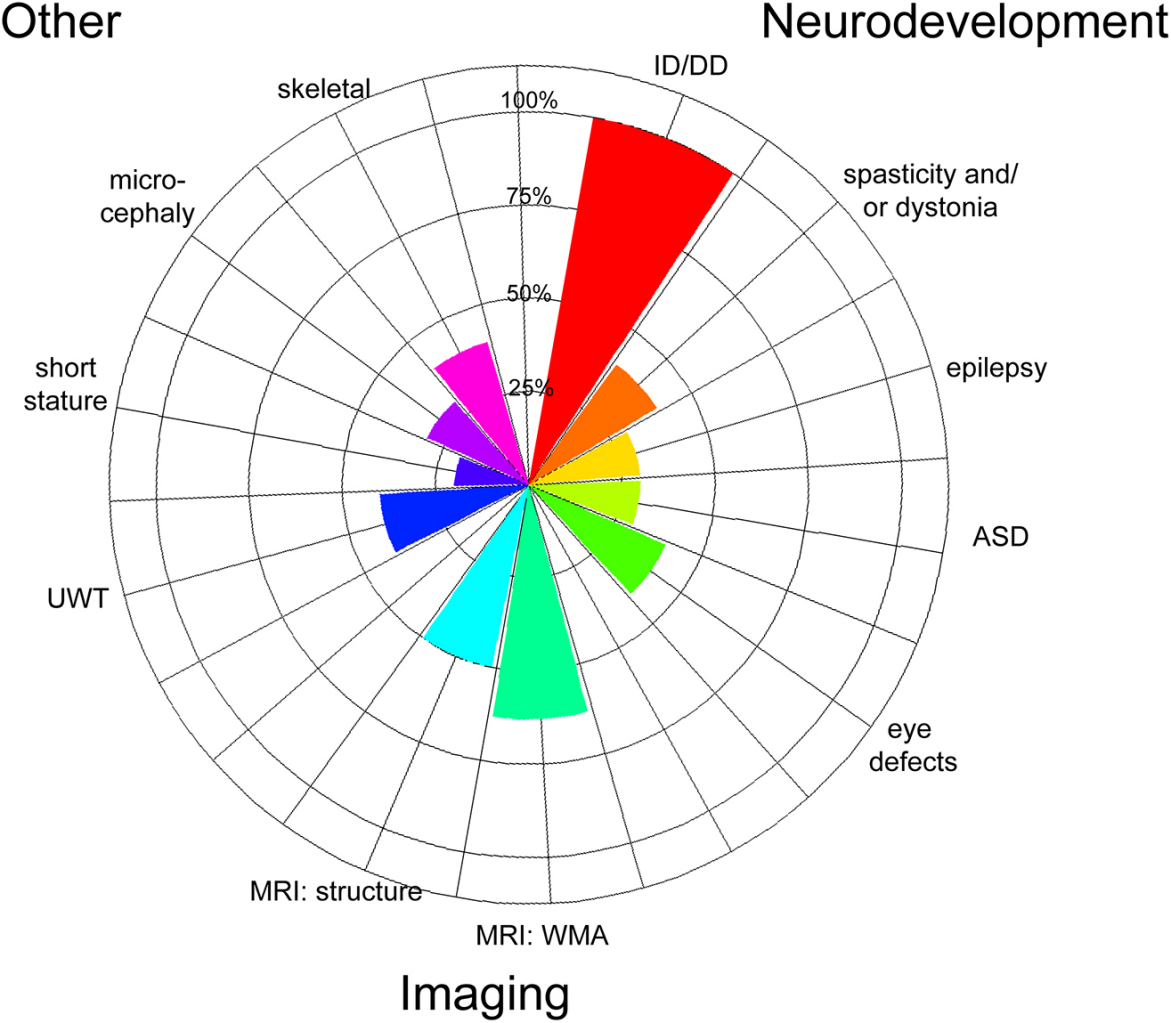
**Phenogram of shared features from ten patients with *AGAP1* variants.** Three patients were identified in this study and seven patients were previously reported to have heterozygous, deleterious variants not present in population variant databases (Exac frequency=0; https://gnomad.broadinstitute.org/) (Table S1). Denominators based on number of patients with phenotype reported in the publication. Magnetic resonance imaging (MRI) findings were calculated using six patients from MRI studies. ‘Structure’ indicates regional loss. ASD, autism spectrum disorder; ID/DD, intellectual disability/developmental delay; UWT, underweight; WMA, white-matter abnormalities.

We identified three new cases in which patients were heterozygous for copy number variants that led to complete or partial *AGAP1* gene deletion (exons 7-9 in one patient and exons 10-13 in another patient) (Table 1). We noted phenotype overlap with seven previously reported cases in which patients had *AGAP1* variants or 2q37 deletions limited to the *AGAP1* region (Table 2; Table S1). Key features include intellectual disability/developmental delay, language impairments, autism spectrum disorder, aggression, and impaired length, weight and cranial growth (Fig. 1). With lower frequency, we also observed epilepsy, dystonia and/or axial hypotonia, brain growth abnormalities, eye abnormalities, and skeletal defects. The shared clinical features of this expanded cohort provide additional support that *AGAP1* variants can lead to a mixed neurodevelopmental disorder with systemic manifestations.

**Table 1.**
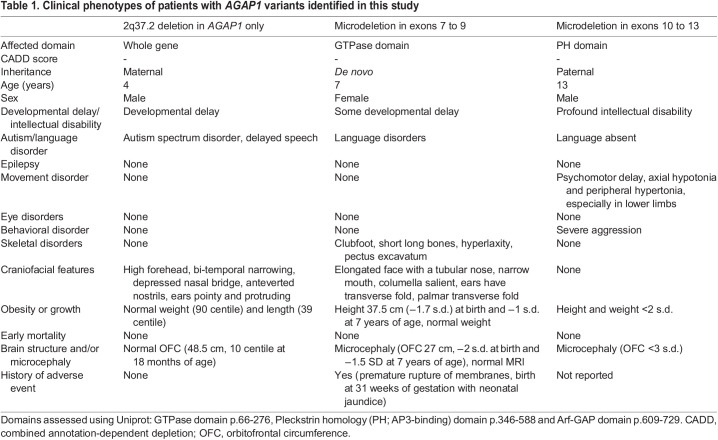
Clinical phenotypes of patients with *AGAP1* variants identified in this study

**Table 2.**
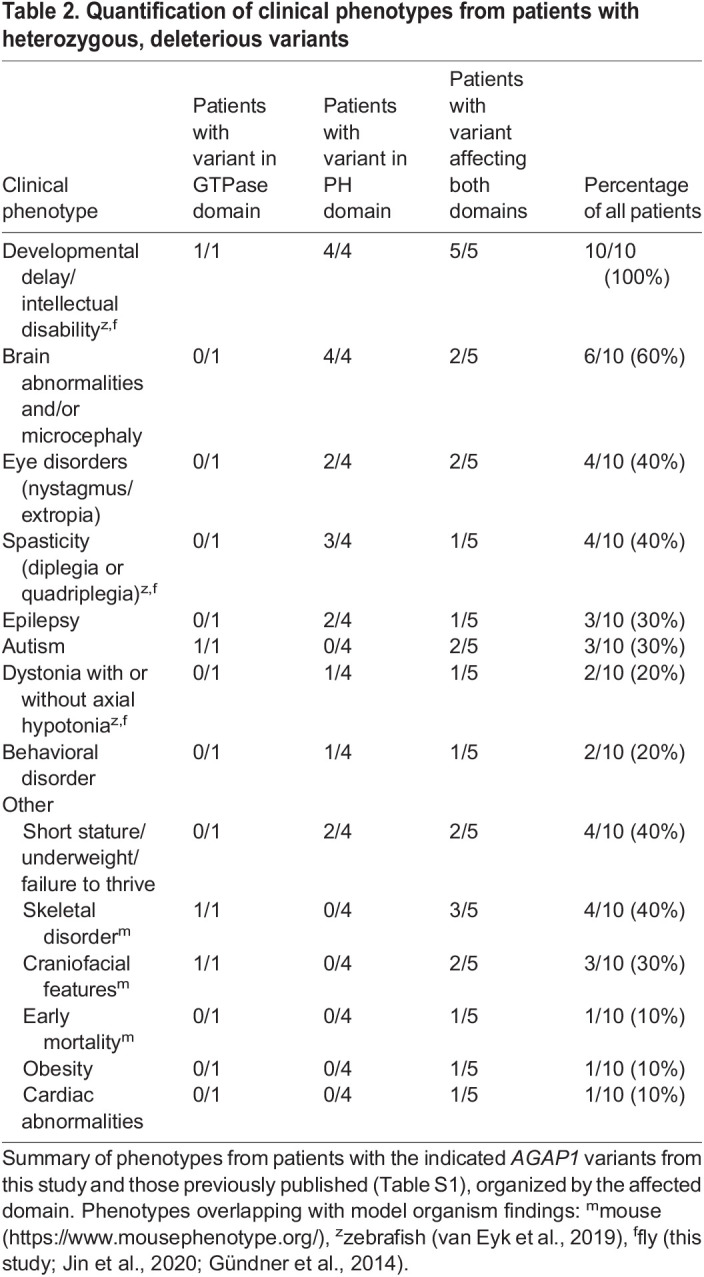
Quantification of clinical phenotypes from patients with heterozygous, deleterious variants

### *CenG1a* is required for scaled growth of the neuromuscular junction

**Fig. 2.**
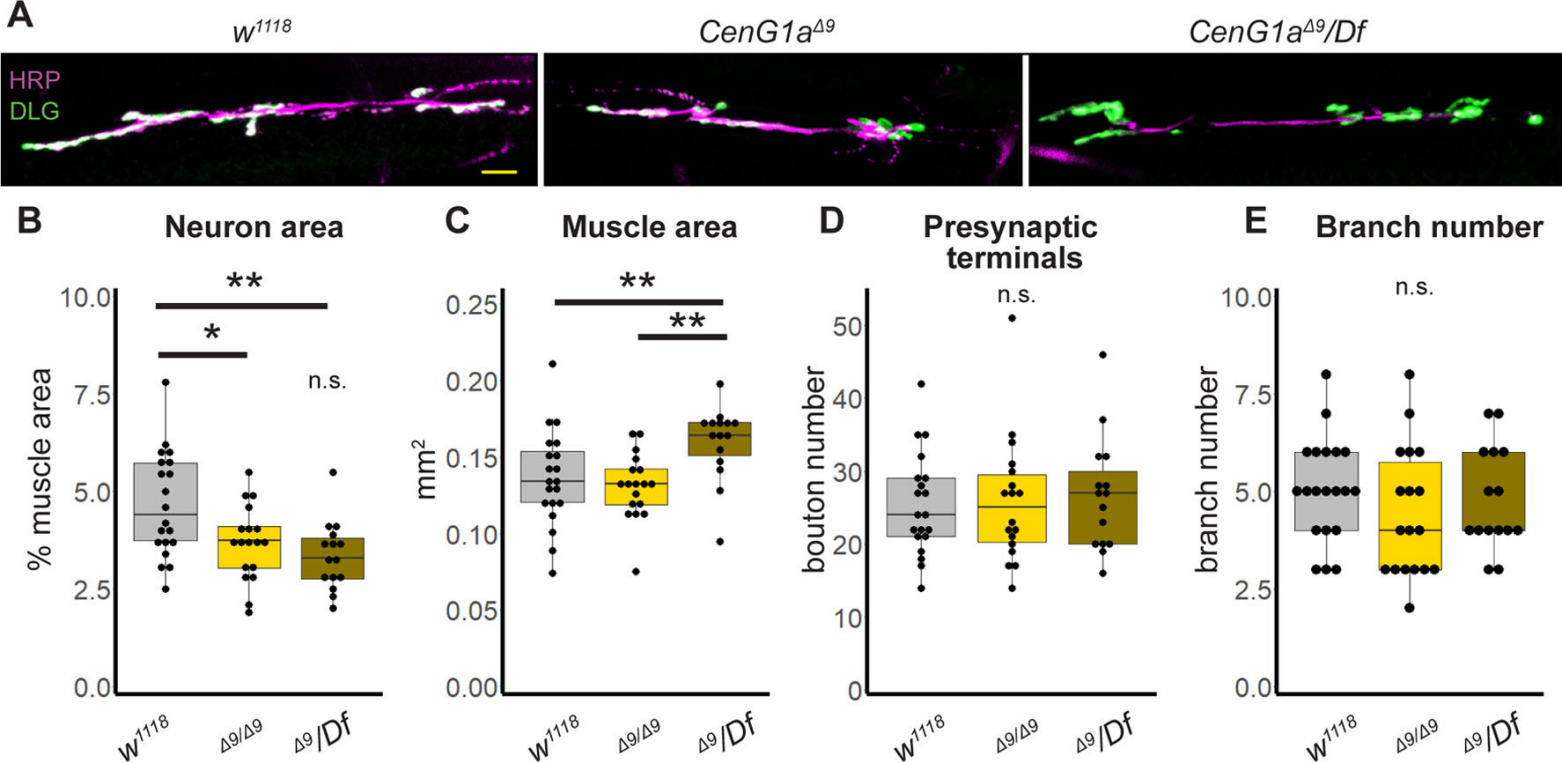
**AGAP1 regulates scaled growth at the *Drosophila* NMJ.** (A) Images of axon terminals from late third instar larva. Horseradish peroxidase (HRP; magenta) marks the neuron membrane and DLG (discs large or Dlg1, psd95 ortholog; green) marks post-synaptic structures to identify individual boutons. Scale bar: 20 µm. (B-E) Box-and-whisker plots of genetic control (*w^1118^*), *CenG1a* null homozygote (*Δ9/Δ9*) and *CenG1a* hemizygote (*Δ9/Df*) to map phenotypes to *CenG1a* genetic locus. Neuronal (HRP-stained) area normalized to muscle area was significantly decreased in both *CenG1a* mutants (B). Muscle area was also increased in hemizygotes, but not in homozygotes (C). The number of boutons, i.e. pre-synaptic terminals (D), and axon branches (E) were not changed in *CenG1a* mutants. *Df* or *Df(2L)BSC252* is a deletion uncovering *CenG1a*; NMJ, neuromuscular junction. Boxes represent the 25th-75th percentiles, whiskers represent the 10th and 90th percentiles, and the median is marked with a line. *w^1118^*: *n*=20 NMJs, 13 animals; *CenG1a^Δ9^*: *n*=18 NMJs, 10 animals; *CenG1a^Δ9^/Df*: *n*=15 NMJs, 10 animals. n.s., not significant; *P<0.05; **P<0.005 (two-tailed Mann–Whitney rank sum test).

We noted movement disorders such as dystonia in patients with *AGAP1* variants (Table 2) and previously showed locomotor impairments in *CenG1a* mutant flies (Jin et al., 2020). Patients with *AGAP1* variants also exhibited microcephaly, impaired cortical growth and delayed myelination, and *AGAP1* is known to be important for dendritic spine maturation (Arnold et al., 2016). We therefore screened *CenG1a* mutants for neuroanatomical phenotypes (Fig. 2A). We utilized a null allele (*CenG1a^Δ9^*), given that AGAP1 putatively acts via loss of function. We found a reduction in the size of the axon terminal (Fig. 2B) without a corresponding decrease in the muscle area in *CenG1a^Δ9^* homozygotes. We confirmed that this was a recessive phenotype arising from loss of *CenG1a* as there was no change in heterozygous animals (Fig. S1) and the phenotype was also present in trans with a deficiency chromosome uncovering *AGAP1* (*CenG1a^Δ9^/Df*)*. CenG1aΔ9/Df* hemizygotes also had an unexpected increase in muscle size (Fig. 2C). We did not detect a change in bouton number or density [bouton number divided by neuromuscular junction (NMJ) area], or the number of satellite boutons (Fig. 2D). Other than the decreased size, neurons appeared morphologically normal with no change in branch number (Fig. 2E). Pre- and post-synaptic marker colocalization was normal (not shown). A degenerative phenotype often has a post-synaptic signal with an absent pre-synaptic signal (Lincoln et al., 2015). In contrast, a synaptic maturation phenotype often has a pre-synaptic signal with an absent post-synaptic signal (Vasin et al., 2014). Given that *CenG1a* mutants only demonstrated reduced synapse size, our findings indicate that this likely represents an impaired neuronal growth or remodeling phenotype rather than representing degeneration or defective synaptogenesis.

### Increase in number and lysosomal localization of Rab7-positive endosomes in motor neurons of *CenG1a* mutants

**Fig. 3.**
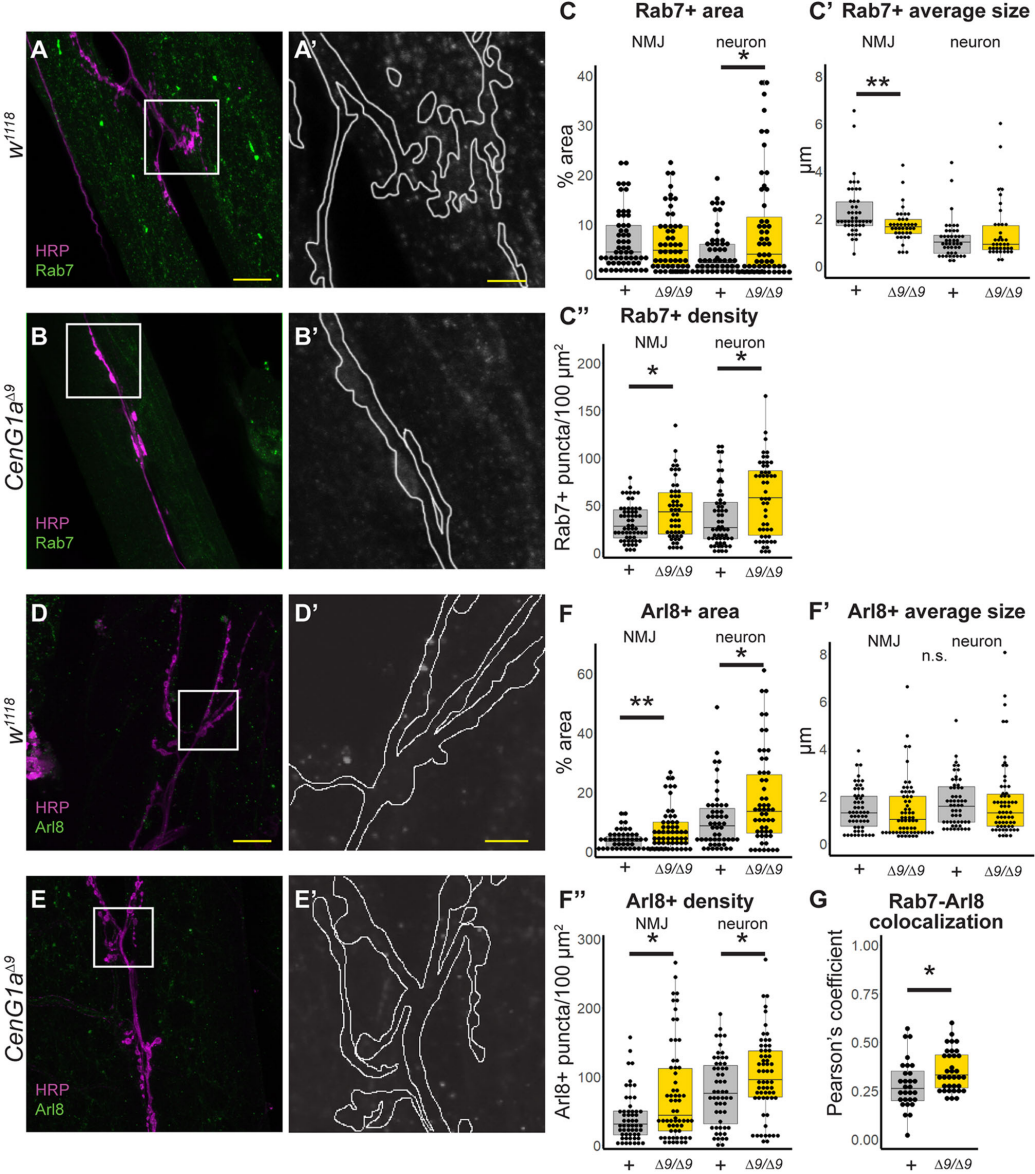
**AGAP1 regulates the endosomal and lysosomal compartments at the *Drosophila* NMJ.** (A,B) HRP (magenta) localizes to neuron membranes and Rab7 (green) is present in early-to-late endosomes of *w^1118^* controls (A) and *CenG1a^Δ9^* null homozygotes (B). Scale bar: 20 µm. (A′,B′) Magnified views of boxes from panels A and B showing the Rab7 channel with the neurons outlined. Notably, more Rab7 staining was present in the outlined neuronal area in *CenG1a*-mutant NMJs (B′) compared to that in controls (A′). Scale bar: 5 µm. (C-C″) Quantification of Rab7 puncta properties. The areas (C) and density (C″) of Rab7 puncta were increased in the neurons of *CenG1a^Δ9^* loss-of-function mutants, but endosome-associated average puncta size (C′) was not. + indicates *w^1118^*, *Δ9* indicates *CenG1a^Δ9^*. *w^1118^*: *n*=58 fields of view (FOVs), 20 NMJs, 12 animals; *CenG1a^Δ9^*: *n*=52 FOVs, 21 NMJs, 10 animals. (D,E) Immunostaining for HRP (magenta) and Arl8 (green), a marker of lysosomes, in controls (D) and *CenG1a^Δ9^* null homozygotes (E). Scale bar: 20 μm. (D′,E′) Magnified views of boxes from panels D and E showing the Arl8 channel with the neurons outlined. Notably, more Arl8 staining is present in the outlined neuronal area of *CenG1a*-mutant NMJs (E′) compared to that in controls (D′). Scale bar: 5 μm. (F) Quantification of Arl8 properties. The lysosome-associated areas (F) and density (F″) were increased in neurons and, to a lesser extent, across the entire NMJ in *CenG1a^Δ9^* mutants, whereas the average size was unaffected (F′). *w^1118^*: *n*=51 FOVs, 23 NMJs, 14 animals; *CenG1a^Δ9^*: *n*=54 FOVs, 25 NMJs, 14 animals. (G) Pearson correlation coefficient was significantly increased between Rab7- and Arl8-positive puncta in *CenG1a^Δ9^* mutant neurons. *w^1118^*: *n*=29 FOVs, 13 NMJs, 8 animals; *CenG1a^Δ9^*: *n*=35 FOVs, 15 NMJs, 8 animals. n.s., not significant; *P<0.05; **P<0.005; (two-tailed Mann–Whitney rank sum test).

AGAP1 is predicted to regulate protein trafficking out of the endosome (Arnold et al., 2016; Nie et al., 2003). We investigated whether *AGAP1* loss of function disrupted trafficking within the endosomal compartment in the nervous system using the *CenG1a* knockout model. We examined the Rab7 marker of early-to-late endosomes (Shearer and Petersen, 2019) in the NMJ (Fig. 3A,B), using an area defined by anti-horseradish peroxidase (HRP) staining to distinguish between axon terminals and muscles. We noted increased Rab7 signal in *CenG1a* mutants (Fig. 3B′) compared to genetic controls (Fig. 3A′), particularly within the HRP-defined neuronal area. The area (Fig. 3C) and density of Rab7-positive puncta (Fig. 3C″) was increased in *CenG1a* mutants with no change in average size (Fig. 3C′) or intensity of signal (not shown). The increased endosome signal was localized to the motor neuron with no change in Rab7 area in the muscle, consistent with nervous system-specific expression of *CenG1a*. Thus, *CenG1a* appears to be important for regulating the number and size of endosomes in neurons.

We investigated whether increased endosome density was due to accumulated endosomes resulting from a failure of macroautophagy, reflecting disrupted endolysosomal degradation. We examined Arl8 (Fig. 3D,E), a marker of lysosomes (Rosa-Ferreira et al., 2018), and noted that its levels were also increased in *CenG1a*-mutant NMJs (Fig. 3E′) compared to its levels in genetic controls (Fig. 3D′). The lysosome-associated area (Fig. 3F) and density (Fig. 3F′) of Arl8 was increased in *CenG1a* mutants, although the average lysosome size was unchanged (Fig. 3F′). We subsequently found that there was increased colocalization between Rab7 and Arl8 puncta in the *CenG1a* mutants (Fig. 3G), reflecting a larger endolysosomal compartment. Taken together, these data demonstrate increases in average lysosome size and content, potentially from increased endosomal fusion (Jacomin et al., 2016), a defect in lysosomal degradation or disrupted endolysosomal reformation (Klionsky et al., 2008).

### *CenG1a* mutants have an increased rate of basal autophagy but normal flux

**Fig. 4.**
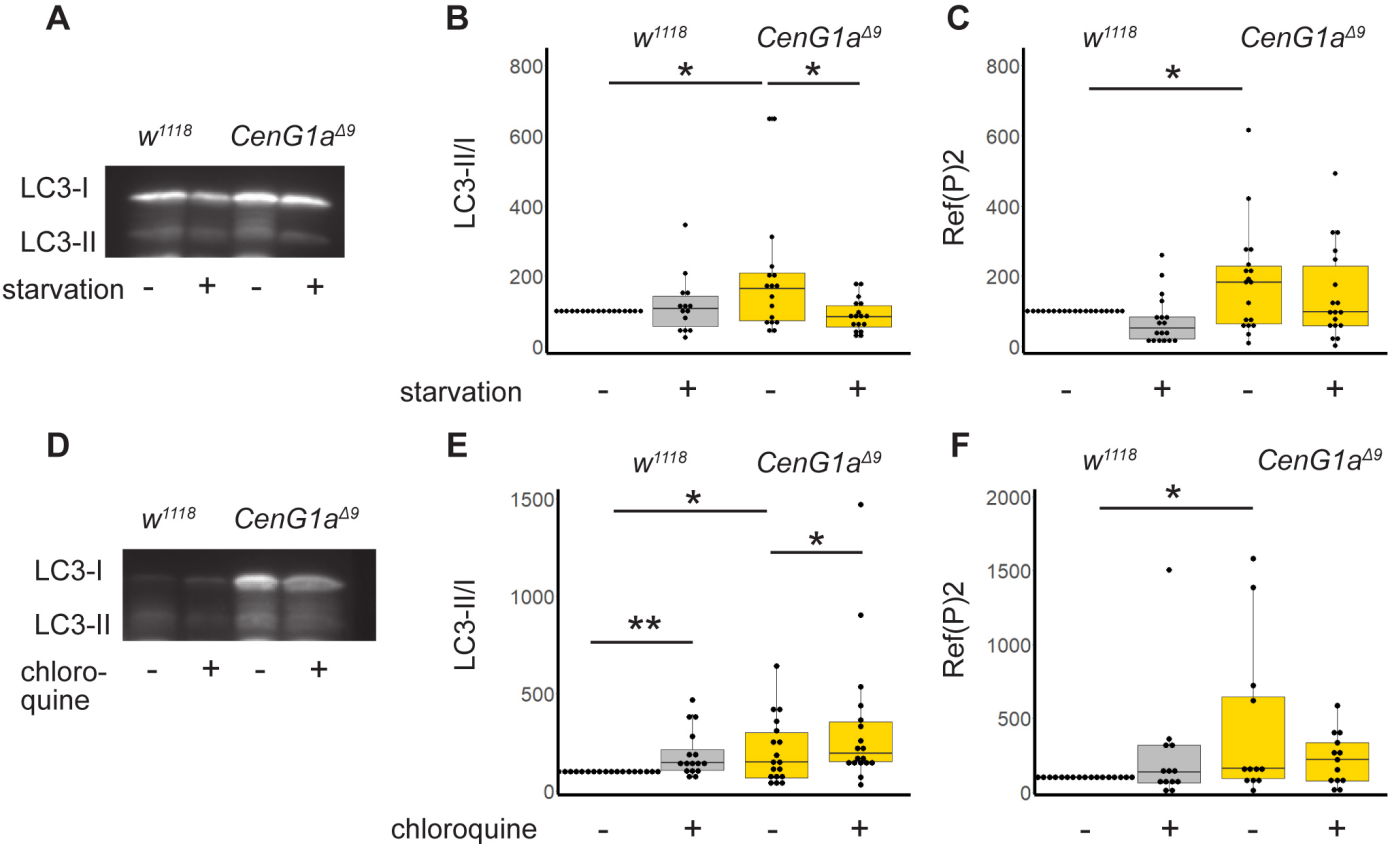
***CenG1a* mutants have increased autophagy induction with normal flux.** (A) Representative western blot of LC3-II and LC3-I (Atg8a) from *w^1118^* controls and *CenG1a^Δ9/Δ9^* homozygous mutants from either fed or starved conditions. (B) Quantification of LC3-II/LC-I ratio from larvae with or without starvation. Values were normalized to those of the *w^1118^* fed condition from the same experiment. Fed *CenG1a^Δ9^* animals had increased LC3-II/LC-I ratios compared to those of fed controls. Control LC3-II/LC3-I ratios did not change after 24 h of starvation, whereas those of *CenG1a^Δ9^* mutants decreased to genetic control levels. *n*=17 from six biological replicates of >20 larvae. (C) Ref(P)2 (*Drosophila* ortholog of p62) levels from larva with or without starvation as analyzed by western blotting. Ref(P)2 levels are elevated in *CenG1a^Δ9^* and do not change during starvation for either genotype. *n*=19 from five biological replicates. (D) Representative blot of LC3-II and LC3-I (Atg8a) with 24 h feeding with vehicle control or chloroquine. (E) Quantification of LC3-II/LC-I ratio from larvae with or without chloroquine feeding to block autophagic flux. *CenG1a^Δ9^* animals had elevated LC3-II/LC-I ratios compared to those of controls. Chloroquine feeding elevated LC3-II/LC-I ratios in both genotypes, demonstrating normal autophagic flux in *CenG1a* mutants. *n*=10 from five biological replicates. (F) Ref2(P) levels were elevated in *CenG1a^Δ9^* mutants compared to those of controls, and levels did not change during 24 h of chloroquine feeding. *n*=17 from six biological replicates. Values were normalized to those of the *w^1118^* vehicle control condition from the same experiment. *P<0.05; **P<0.005 (two-tailed paired *t*-test). All other comparisons were not significant.

We next asked whether the increased endosomal–lysosomal colocalization was owing to impaired autophagic flux or increased autophagy in *CenG1a^Δ9^* mutants. We examined Atg8a, the *Drosophila* ortholog of LC3, which is lipidated in order to target membranes to the autophagosome. This modification is detected as a size shift in western blotting and calculated as lipidated/basal (LC3-II/LC3-I) levels. We found that *CenG1a* mutants had a higher ratio of LC3-II/LC3-I compared to the baseline in genetic controls (Fig. 4A,B). We induced starvation to assess the ability of *CenG1a^Δ9^* mutants to respond to an exogenous cytotoxic stressor. LC3-II/LC3-I ratios did not change in genetic controls after 24 h of starvation, indicating optimized balance between autophagy induction and protein degradation and clearance. In contrast, starvation decreased the basally elevated LC3-II/LC3-I ratios in *CenG1a* mutants.

We then tested whether autophagic flux was normal by treating larvae with chloroquine (Zirin et al., 2013), a weak base that blocks autophagy by altering the acidic environment of lysosomes and autophagosome–lysosome binding. We found that chloroquine feeding increased the ratios of LC3-II/LC3-I for both genetic controls and *CenG1a^Δ9^* mutants, as would be expected when blocking normal autophagic flux (Fig. 4D,E). This also suggests that lysosomal function is unaffected in *CenG1a* mutants.

We then assessed Ref(2)P, the *Drosophila* ortholog of p62 (SQSTM1), which recruits ubiquitinated cargos to autophagosomes; p62 accumulates when autophagy is impaired (Pircs et al., 2012). Consistent with elevated LC3-II/LC-I ratios, we found increases in Ref(2)P in *CenG1a^Δ9^* larvae compared to its levels in genetic controls (Fig. 4C,F). We did not detect a significant difference in Ref(2)P within genotypes between fed and starved conditions, nor with chloroquine treatment at 24 h. Taken together, our data demonstrate higher levels of autophagy induction in *CenG1a* mutants compared to the baseline in controls, but with preserved flux and increased clearance during starvation.

### *CenG1a* mutants have a diminished capacity to respond to additional stressors via eIF2α phosphorylation

**Fig. 5.**
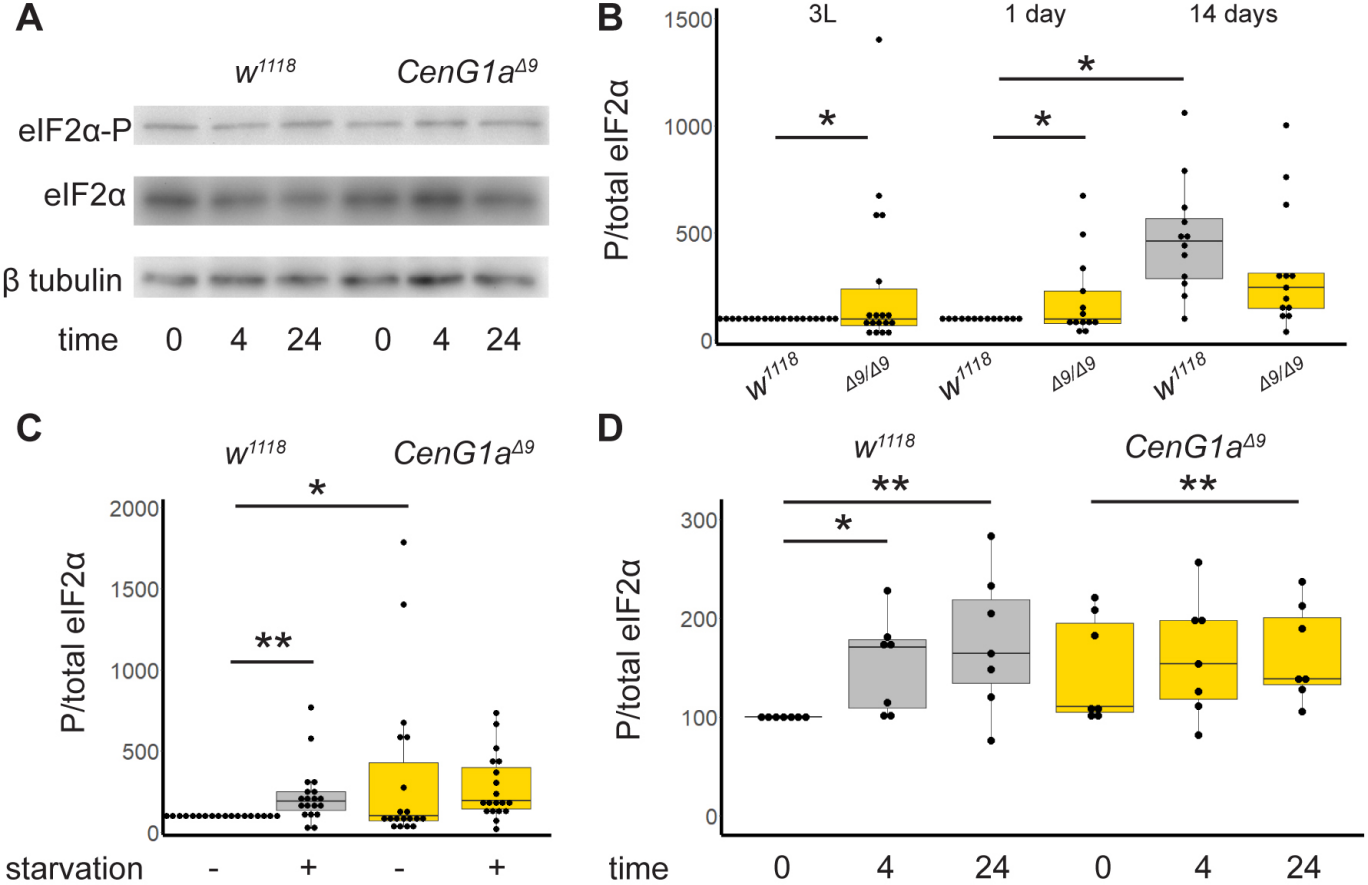
***CenG1a* mutants have elevated levels and defective regulation of eIF2α phosphorylation.** (A) Representative western blots from 3-day-old adults exposed to tunicamycin for 0, 4 or 24 h, cropped to show phosphorylated eIF2α (eIF2α-P), and then the same blots were reprobed for total eIF2α and β-tubulin levels. Quantification is shown in D. (B) eIF2α-P levels of *w^1118^* (grey) and *CenG1a^Δ9^* homozygous (gold) third instar larvae (3L, left), 1-day-old (center) and 14-day-old (right) adults. Control animals had a significant increase in eIF2α-P from 1 to 14 days post eclosion. In contrast, *CenG1a* mutants did not show altered eIF2α-P levels with age and had reduced eIF2α-P levels compared to those of wildtype controls at 14 days post eclosion. *n*=14 from five biological replicates for larva and *n*=13 from three biological replicates for adults. (C) eIF2α-P levels of second instar larva either deprived of yeast (starved) or fed for 24 h before protein extraction. Wild-type flies showed increased eIF2α-P levels in response to starvation stress, whereas *CenG1a^Δ9^* mutants had elevated eIF2α-P levels that did not change in response to starvation. *n*=14 from five biological replicates. (D) Adults 3 days post eclosion were moved to vials with tunicamycin-containing food (12 µM) for 0, 4 or 24 h before protein extraction. Control animals showed increased eIF2α-P levels in response to tunicamycin-induced unfolded protein stress at 4 and 24 h. In contrast, eIF2α-P levels in *CenG1a^Δ9^* mutants were equivocally elevated compared to those of controls (*P*=0.06) and had a delayed response wherein they did not increase until 24 h of exposure. *n*=7 from two biological replicates third instar larva. Values were normalized to those of the *w^1118^* no treatment condition from the same experiment. *P<0.05; **P<0.005 (two-tailed paired *t*-test). All other comparisons were not significant.

Disruptions of protein trafficking (Amodio et al., 2019) and clearance (Xiong et al., 2013) have been linked to activation of the integrated stress response. When the α subunit of the eukaryotic initiation factor-2 (eIF2α or eIF2A) is phosphorylated, global protein translation decreases, whereas the expression of stress response genes and autophagy components is selectively upregulated (B'chir et al., 2013; Humeau et al., 2020). We therefore investigated whether loss of *CenG1a* altered eIF2α phosphorylation (eIF2α-P) (Fig. 5A).

We found a significant increase in eIF2α-P in *CenG1a^Δ9^* larvae and young, 1-day-old adults (Fig. 5B). Control adults showed increased eIF2α-P between 1 and 14 days post eclosion, consistent with previous descriptions of normal aging (Lenox et al., 2015). In contrast, *CenG1a^Δ9^* animals showed elevated eIF2α-P early in development, but mutant animals did not show an age-dependent upregulation of eIF2α-P over time. These results indicate that eIF2α phosphorylation is chronically activated in mutant animals.

We next examined whether basal elevations in autophagy and eIF2α-P impaired responses to stress in a second-hit scenario. Amino acid starvation normally activates phosphorylation of eIF2α through the GCN2 kinase (Pakos-Zebrucka et al., 2016). When challenged with 24 h of starvation, control larvae exhibited increased eIF2α-P levels. In contrast, *CenG1a^Δ9^* larva with chronically elevated eIF2α-P levels did not show a further increase in its levels (Fig. 5C). This indicates a diminished capacity to respond to a second-hit cytotoxic stressor.

This suggested to us that *CenG1a^Δ9^* animals may have increased sensitivity to exogenous stressors. Accordingly, we exposed adult flies to tunicamycin, which blocks protein glycosylation. Tunicamycin impairs protein folding, induces the integrated stress response in the endoplasmic reticulum (ER), and triggers phosphorylation of eIF2α via PERK kinases (EIF2AK3) (Chow et al., 2013). Control animals exhibited increase eIF2α-P at 4 and 24 h of tunicamycin exposure. In contrast, eIF2α-P was equivocally upregulated in *CenG1a*-mutant adults compared to baseline levels in controls (*P*=0.06). In this context, *CenG1a* mutants exhibited a delay in their activation of stress response pathways, as there was a failure to increase eIF2α-P at 4 h, although phosphorylation was increased after 24 h (Fig. 5D). Thus, multiple stress response pathways are chronically active in *CenG1a* mutants and do not respond appropriately to acute stressors.

**Fig. 6.**
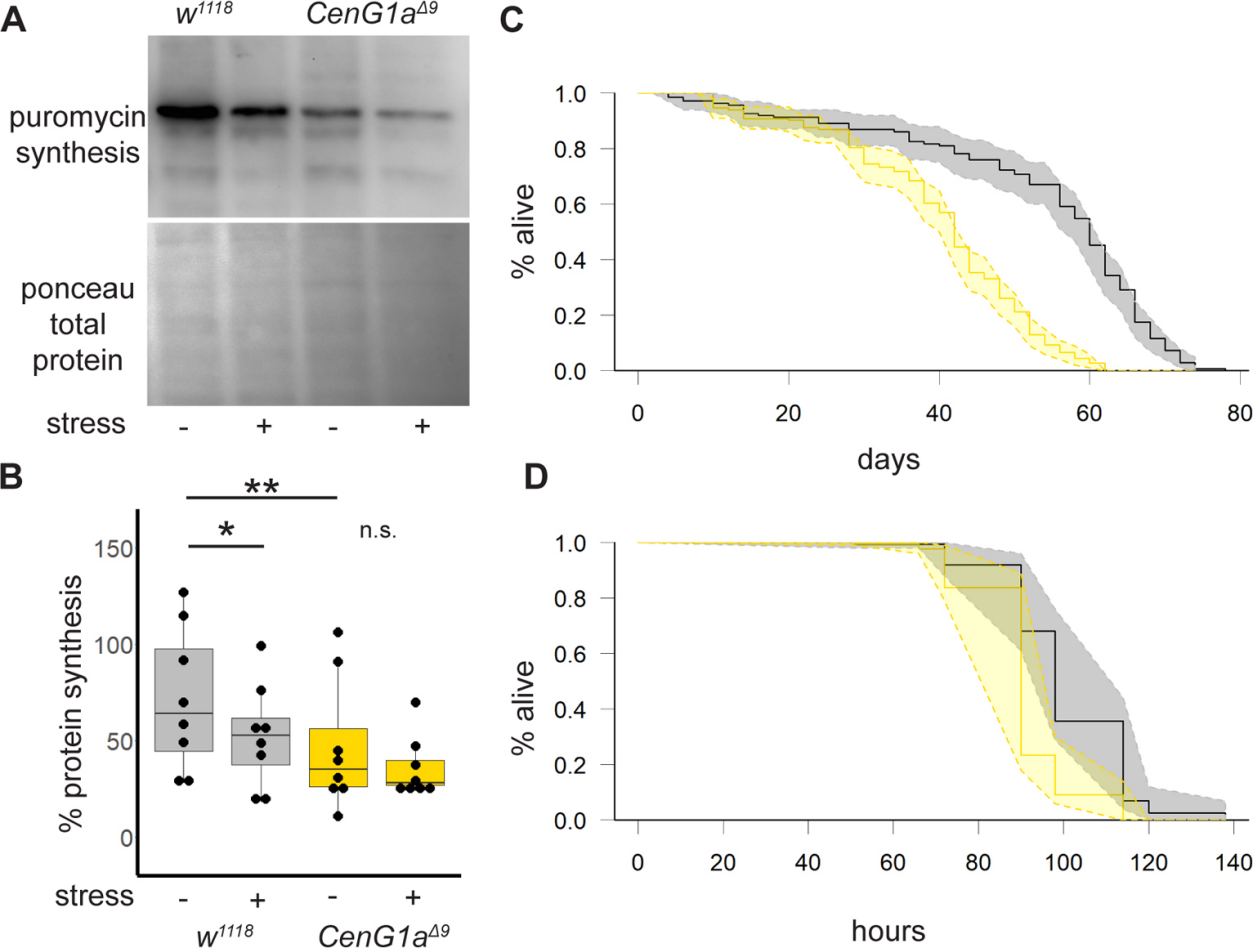
**Decreased protein translation and sensitivity to stress in *CenG1a* mutant flies*.*** (A) Newly synthesized proteins identified by puromycin incorporation and western blotting with anti-puromycin antibody. ER stress was induced by adding 12 μM tunicamycin to tissue incubation solution. (B) Quantification of the ratio of the in-lane puromycin signal divided by Ponceau-labeled signal, indicating the percentage of protein synthesis. *n*=8 from four biological replicates. n.s., not significant; *P<0.03; **P<0.008 (two-tailed Mann–Whitney rank sum test). (C) *CenG1a^Δ9^* homozygous adult flies had reduced lifespan. Kaplan–Meier survival curves with 95% confidence interval shaded between dashed lines. *w^1118^*: *n*=149 flies, 15 vials; *CenG1a^Δ9^*: *n*=184 flies, 15 vials. *P*<2×10^−16^ (log-rank test). (D) *CenG1a^Δ9^* flies were more sensitive to tunicamycin lethality. Significantly fewer *CenG1a^Δ9^* flies survived between 90-114 h from the start of the treatment. No lethality in DMSO control vials was observed during this time. *w^1118^*: *n*=146 flies, 13 vials; *CenG1a^Δ9^*: *n*=139 flies, 13 vials. *P*<5×10^−16^ (log-rank test).

We confirmed that elevated eIF2α-P has functional consequences on the rates of protein synthesis with puromycin labeling. As expected, control larvae exhibited decreased global protein synthesis in the presence of tunicamycin-induced ER stress. In contrast, *CenG1a^Δ9^* mutants exhibited decreased levels of protein synthesis compared to baseline levels in controls and failed to further reduce the levels of protein synthesis in the presence of this stressor (Fig. 6A,B).

We next investigated whether impaired eIF2α-P creates functional impairments to stress responses in *CenG1a* mutants. Longevity studies revealed that *CenG1a* mutants died earlier than their genetic controls under normal rearing conditions (Fig. 6C). We sought to understand whether the inability of mutant animals to respond to a subsequent cytotoxic stressor would have important consequences at the level of the whole organism. We found that *CenG1a* mutants were more sensitive to ER stress-induced lethality, with tunicamycin-treated animals dying much earlier than genetic controls (Fig. 6D). Our findings illustrate a two-hit scenario wherein variants in *CenG1a* increases the susceptibility of animals to a subsequent insult.

## DISCUSSION

We found evidence for an expanded endolysosomal compartment in *CenG1a* mutants. Accordingly, we also identified increased autophagy and eIF2α phosphorylation under basal conditions, suggesting chronic activation of the cytotoxic stress response. Autophagic flux was preserved in mutants, suggesting that lysosomal function was unaffected, but mutant animals had a decreased capacity to respond to ‘second-hit’ cytotoxic stressors. When challenged with such a stressor, mutants exhibited diminished survival.

Heterozygous deletions and predicted damaging missense variants in *AGAP1* are associated with a mixed neurodevelopmental phenotype that includes autism spectrum disorder and cerebral palsy. *AGAP1* is predicted to regulate AP3 trafficking proteins via the PH domain and patient phenotypes overlap with those caused by variants in the gene encoding an AP3-complex member, *AP3D1. AP3D1* variants cause Hermansky–Pudlak syndrome 10; a patient with this syndrome shared several features with patients with *AGAP1* variants, including global developmental delay, epilepsy, dystonia, hypotonia, poor feeding, diminished cortical volumes and poor myelination detected by neuroimaging, and microcephaly (Ammann et al., 2016). Consistent with the report on one patient with an *AGAP1* variant (Jin et al., 2020), the patient with the *AP3D1* variant had immunodeficiency and died of septic pneumonia at the age of 3.5 years. Disruptions to endosomal trafficking, such as to the retromer complex, are increasingly being linked to developmental disease, including hereditary spastic paraplegia and Ritscher–Schinzel Syndrome, with features spanning neurological, skeletal and immune systems (Saitoh, 2022; Yarwood et al., 2020).

We found decreased synaptic size in *Drosophila* with homozygous null variants in *CenG1a*, which was not apparent in a previous study decreasing *CenG1a* expression using RNAi knockdown or P-element gene disruption (Homma et al., 2014). We observed this phenotype in animals with a homozygous null allele or in trans with a deficiency, but not in heterozygotes. This suggests that the synaptic size phenotype is AGAP1 dependent, but with haploinsufficient inheritance of microcephaly in humans and recessive inheritance of NMJ size defects in the fly. This has been observed in related disorders (Gatto and Broadie, 2011; Tsai et al., 2012). It is therefore possible that the increased synaptic release found by Homma et al. (2014) may represent a mechanism to compensate for *CenG1a* partial loss of function to maintain synaptic function. We did not test whether AGAP1-mediated Arf1 activity or actin cytoskeleton regulation could underly the axon terminal size phenotype. Thus, the molecular mechanism by which *AGAP1* leads to decreased axon terminal size needs further investigation. It is unlikely that decreased axon terminal size or increased synaptic release is due to increased autophagy; increased autophagy is associated with increased synaptogenesis in *Drosophila* and decreased synaptic release (Shen et al., 2015). Therefore, we conclude that the autophagy increase we observed compared to baseline levels is likely to be a compensatory mechanism.

In addition to an increased rate of basal autophagy, we observed an increase in basal eIF2α phosphorylation. Both indicate an activation of stress response pathways in the absence of external provocation. This argues that this basal activation is instead triggered in response to endogenous factors – the *CenG1a* genetic variant and the dysregulation of endolysosomal trafficking that ensues.

The activation of these elements of the integrated stress response likely serves as a compensatory mechanism; however, this mechanism is saturable. This sets up the potential sensitivity that leaves *CenG1a* mutants with a diminished capacity to respond to additional cytotoxic stressors, such as amino acid deprivation, tunicamycin or aging, and is observed here as increased mortality. Consistent with the idea of variants in *AGAP1* increasing sensitivity to stress, we noted that one of the patients in this cohort had a history of prematurity, which may have also contributed to their disorder. We also noted that two patients inherited variants from a healthy parent. We further identified 4/7 cerebral palsy patients with *AGAP1* variants from previously published studies who also had an adverse event in their medical history (Table S1). This further suggests that AGAP1 may contribute to disease in a gene–environment interaction by increasing sensitivity to diverse stressors in some cases. Our findings indicate that the gene–environment interaction is not limited to perturbations of protein trafficking, as we showed that starvation and ER stress both elicit phenotypes. The integrated stress response may thus represent a point of molecular convergence that connects several forms of genetic and environmental insults. Although this will require additional experimental validation, we suspect that this phenomenon may be broadly applicable to a variety of gene–environment interactions that contribute to human neurodevelopmental disorders and is not unique to *AGAP1* variants.

## MATERIALS AND METHODS

### Patient recruitment and data collection

Human subjects oversight for this study was provided by Phoenix Children's Hospital's Institutional Review Board #15-080. All participants (affected children and their parents) provided express written informed consent for participation in accordance with local rules and regulations as specified in the Declaration of Helsinki. Participants with variants in *AGAP1* were identified through GeneMatcher (Sobreira et al., 2015) and de-identified phenotypic data were shared after local whole-exome sequencing and/or microarrays were performed. 2q37.2 microdeletion limited to *AGAP1* was detected by microarray. Microdeletions were identified by array comparative genomic hybridization (Human Sureprint 2×105K, Agilent Technologies) and confirmed with semiquantitative PCR.

### *Drosophila* genetics and rearing

*Drosophila melanogaster* were reared on a standard cornmeal, yeast, sucrose food from the BIO5 media facility, University of Arizona. Stocks for experiments were reared at 25°C, 60-80% relative humidity with 12 h:12 h light/dark cycle. Cultures for controls and mutants were maintained with the same growth conditions, especially the density of animals within the vial. A mix of males and females were used for all experiments.

*CenG1a^Δ9^* is a null allele generated by ends-out gene targeting (Gündner et al., 2014) and was a kind gift from Michael Hoch (Department of Molecular Developmental Biology, University of Bonn, Life & Medical Sciences Institute). *Df(2L)BSC252*, *w^1118^* and Canton-S were acquired from Bloomington *Drosophila* Stock Center [National Institutes of Health (NIH), P0OD018537]. *CenG1a^Δ9^* was backcrossed with *w^1118^* for three generations and *w^1118^* was outcrossed with Canton-S and backcrossed with *w^1118^* for 12 generations while selecting for red eyes.

### Immunohistochemistry and imaging at the NMJ

Imaging of NMJs of third instar wandering larvae was performed as previously described (Estes et al., 2011). Briefly, a larval fillet was dissected in HL-3 Ca^2+^-free saline (70 mM NaCl, 5 mM KCl, 22 mM MgCl_2_, 10 mM NaHCO_3_, 5 mM trehalose, 115 mM sucrose, 5 mM HEPES, pH 7.3) before fixation in Ca^2+^-free 4% paraformaldehyde. Washes were performed with PBS without or with 0.1% Triton X-100 (PBST). Blocking before and during primary and secondary antibody steps used PBST with 5% normal goat serum and 2% bovine serum albumin. The following primary antibodies [Developmental Studies Hybridoma Bank (DSHB) hybridoma monoclonal antibodies; details in Table S2] were incubated overnight at 4°C: mouse anti-Rab7 (1:10; deposited by S. Munro), rabbit anti-Arl8 (1:200; deposited by S. Munro) and mouse anti-DLG (1:400; 4F3; deposited by C. Goodman). Secondary antibodies were incubated for 1.5 h at room temperature and included: goat anti-mouse Cy3-conjugated IgG, goat anti-rabbit Cy3-conjugated IgG or donkey anti-rabbit Alexa Fluor 488-conjugated IgG (1:400; Thermo Fisher Scientific). Neuronal membranes were visualized with goat anti-HRP-Alexa Fluor 647 (1:100; Jackson ImmunoResearch) added with secondary antibody. Phalloidin-488 in PBS (1:300; Molecular Probes) was added as the final wash before mounting.

Imaging was performed with a 710 Zeiss confocal microscope using 1.0 µm *z*-stacks at 63× of the 1b 6/7 muscle in the A3 abdominal segment. Image parameters (laser gain and intensity, resolution, zoom) were kept constant between images of the same session; alternating between those for the control and mutants. Maximum-intensity projections were created for a given field of view using all images of the *z*-stack that included the axon terminal (defined by HRP staining) for all channels. Projections were converted to greyscale from RGB in Photoshop without adjustments to pixel intensity. Non-6/7 1b-HRP staining was identified based on the shape, size and HRP staining intensity and manually removed.

Images were analyzed using ImageJ software v1.50i (NIH). Images from individual channels were converted to black and white using the threshold feature with the threshold held uniform for images within an imaging session. Rab7 and Arl8 areas were measured using the ‘Analyze Particles’ tool with a mask created from the HRP image to define and measure the neuron-only area. NMJ area was measured by drawing around the area encompassed by both muscles and neuron in the field of view. Pearson correlation coefficient was determined using the coloc2 plugin from Fiji using the HRP channel to provide a mask for Rab7 and Arl8 channels. Orthogonal projections were converted to 8-bit black-and-white TIFFs without thresholding. Boutons were counted manually from 63× images stitched over the entire neuron area. Statistical analyses were performed and graphs created using R v4.1.0. For boxplots, boxes represent the 25th-75th percentiles, and whiskers represent the 10th and 90th percentiles.

### Western blotting and quantification

Protein samples were prepared from ten to 20 males and females in protein extraction buffer plus 1% protease inhibitor (Thermo Fisher Scientific) and 1% phosphatase inhibitor (Sigma-Aldrich) as previously described (Emery, 2007). Western blotting was performed according to standard methods with detection on a 0.2 µm nitrocellulose membrane; antibodies are detailed in Table S2. For eIF2α, the phosphorylation-specific antibody was imaged first, then the membrane was washed and reprobed for total eIF2α. The following antibodies were used to detect autophagy: rabbit anti-Ref(2)P (1:500; Abcam, 178440) normalized using Ponceau staining (Santa Cruz Biotechnology, sc-301558), or mouse anti-β-actin (1:2000; Abcam, 8224) and rabbit anti-Atg8 (1:2000; Sigma-Aldrich, ABC974). The following antibodies were used to detect eIF2α-P: rabbit anti-phospho-S51 (1:1000; Cell Signaling Technology, 3597) and rabbit anti-eIF2S1 (1:500; Abcam 4837). Newly synthesized proteins were labeled with mouse anti-puromycin (1:1000; Kerafast, EQ0001) and their levels normalized using Ponceau staining. The following secondary antibodies were used: goat anti-rabbit or goat anti-mouse ECL IgGs (1:10,000; GE Healthcare, NA931 and NA934).

Chemoluminescence was captured using a FluorChem Imager (Biotechne) and quantified with Image studio Lite (LI-COR). Ref(2)P levels, LC3-II/LC3-I ratio and phosphorylated/total eIF2α ratio were normalized to those for wild-type or no treatment control for each biological replicate. Areas of the same size were used to measure total protein and puromycin signal for each lane to reduce variability. At least two technical replicates of each sample were used for quantification. Differences between genotypes were calculated using two-tailed paired *t*-test and graphs were generated using R v4.1.0. Detailed statistics are provided in Table S3.

### *Drosophila* stress paradigms and drug treatment

For tunicamycin exposure, *w^1118^* and *CenG1A^Δ9^* male and female adults at 3 days post eclosion were anesthetized on ice and pooled into vials filled with 1.3% agar, 1% sucrose and 12 µM tunicamycin (Sigma-Aldrich), with a subset retained for immediate protein extraction. Flies were anesthetized on ice at 4 and 24 h for protein extraction.

Larvae were collected as second instar larvae and washed in water. For larval starvation, >20 male and female larvae were added to petri dishes made with 1.3% agar and 1% sucrose, with or without baker's yeast supplement, and returned to the 25°C incubator. Larvae were collected after 24 h; immobile or pupated animals were excluded. Chloroquine-treated food was prepared as described by Zirin et al. (2013) at a concentration of 3 mg/ml (Sigma-Aldrich). Larvae were collected as above and placed on food mixed with either chloroquine or water as a control for 24 h and then collected for protein extraction. Successful uptake of the drug was confirmed by examining larval gut for Bromophenol Blue that had been mixed into the food.

Puromycin labeling performed as described by Deliu et al. (2017). Briefly, third instar larva were floated out of food with 20% sucrose, washed in water, and inverted in batches of 15-20 mixed males and females. Larvae were incubated in Schneider's insect medium (Thermo Fisher Scientific) with either 12 μM tunicamycin or DMSO (Sigma-Aldrich) control for 3 h at 25°C. Then, 5 µg/ml of puromycin (Thermo Fisher Scientific) was added and incubated at 25°C for 40-60 min before protein extraction.

### Adult survival

*Drosophila* were collected as late-stage pupae, separated by sex, and sequestered in standard food vials with ∼20 animals/vial. The numbers of live animals were monitored daily, with animals transferred to fresh food-containing vials once per week. For tunicamycin lethality, adults were sequestered in vials with 1.3% agar and 1% sucrose with 12 µM tunicamycin or DMSO as a control and monitored twice daily (Chow et al., 2013). Survival library in R v4.1.0 was used to generate Kaplan–Meier plots and log-rank test statistics.

## Supplementary Material

10.1242/dmm.049838_sup1 Supplementary information
