# Supplementary material for: AGAP1-associated endolysosomal trafficking abnormalities link gene–environment interactions in neurodevelopmental disorders
Source: Dis Model Mech. 2023 Sep 26;16(9):dmm049838. doi: 10.1242/dmm.049838 (PMC10548112; doi:10.1242/dmm.049838)
Supplement: Supplementary information [file dmm-16-049838-s1.pdf]

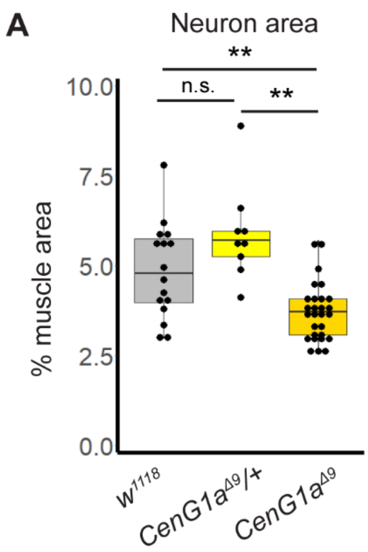

Fig. S1. AGAP1 Drosophila NMJ scaled growth phenotype is recessive.

A. Box and whisker plots of genetic control (*w*^{1118}), *Ceng1a* heterozygote (*CenG1a*$^{\Delta 9}$/+), and null homozygote (*CenG1a*$^{\Delta 9}$). Neuron (HRP) area normalized to muscle area is significantly decreased in *CenG1a* mutants compared to both the genetic control and heterozygote, while there is no difference between heterozygote and genetic control. ** p< 0.0008 by t-test. *w*^{1118} n=16, *CenG1a*$^{\Delta 9}$/+ n=9, *CenG1a*$^{\Delta 9}$ n=28.

Table S1. Phenotypes from previously reported AGAP1-patients. ASD=autism spectrum disorder; CP=cerebral palsy; DD=developmental delay; ID=intellectual disability; PVL=periventricular leukomalacia; TTTS=twin-to-twin transfusion syndrome.

Variant	Domain	Inheritance	Patient features	Patient History	Ref.
c.1400C>G p.P467R	PH	De novo	DD, spastic diplegic CP, dystonia, MRI: PVL, hypogenetic corpus callosum w/ absent splenium.	born 28 weeks, TTTS	van Eyk et al. 2019
c. 1232C>T p.P411L	PH	Not maternally inherited	DD, epilepsy, spastic quadriplegic CP, nystagmus, feeding difficulties, cholestasis, MRI intraventricular haemorrhage, PVL with cystic cavities	Prematurity, intraventricular haemorrhage	van Eyk et al. 2019
c.1462G>A p.Asp488Asn	PH	Not maternally inherited	spastic diplegic CP; postnatal microcephaly; exotropia; optic nerve atrophy (resolved); global DD; failure to thrive; multiple hyperpigmented macules; MRI:	hypoxic ischemic encephalopathy	Chopra et al. 2022

Disease Models & Mechanisms • Supplementary information

			diffuse hypoxic injury		
c.957+1G>A splicing	both	De novo	ID/DD, epilepsy, swallowing issues, underweight, short stature, generalized hypotonia, dystonic posturing, myoclonic jerks, spastic quadriplegic CP, early mortality from pneumonia, MRI; PVL, basal ganglia regions missing	Emergency caesarean with APGAR scores 1@ 1 min, 7 @ 5 min	Jin et al., 2020
deleted region Chr2:235670886–236827204	both	Not available	mild global DD, obesity, retrocerebellar cyst	Not available	Leroy et al., 2013
deleted region chr2:234966458-236025317	both	De novo	ID, ASD, nystagmus, delayed global milestones, aggressive behavior, stereotypic patterns, sleeping disorder, low weight	born full term after an uneventful pregnancy	Pacault et al., 2019

Disease Models & Mechanisms • Supplementary information

deleted region chr2:233854244-243068396	both	De novo	DD/ID, mild thoracic kyphosis and lumbar lordosis, small ears, hypertelorism, short upturned nose with depressed nasal tip, brachydactyly, sinus tachycardia, renal calcification left kidney and small ectopic right kidney, significant anxiety	None	Decipher 333250 Firth et al., 2009

Disease Models & Mechanisms • Supplementary information

Table S2. Antibodies and biological reagents used in studies

Additional biologicals: Normal goat serum Abcam ab7481 lot GR3188160

Schneider's insect media (Thermo) 21720024 lot 1894706

Antibody/stain	Species	Concentration	Application	Company	Lot(s) #
Rab7	M	1:10	NMJ IHC	DSHB	9-26-16
Arl8 polyclonal supernatent	R	1:200	NMJ IHC	DSHB	6/9/16
DLG (4F3)	M	1:400	NMJ IHC	DSHB	2/18/16
Anti-mouse Cy3	G	1:400	NMJ IHC	ThermoFisher	A10521 1425618
Anti-rabbit Cy3	G	1:400	NMJ IHC	ThermoFisher	A10520 1633861
Anti-rabbit 488	D	1:400	NMJ IHC	ThermoFisher	A11008 1515529
Anti-HRP 647	G	1:100	NMJ IHC	Jackson ImmunoResearch	126323
Phalloidin 488	-	1:300	NMJ IHC	Molecular Probes	1903540 2160010
Ref(2)P	R	1:500	WB	Abcam 178440	GR3389640
Beta actin	M	1:2000	WB	Abcam 8224	GR14272-3
Beta tubulin	R	1:2000	WB	Abcam 6046	GR3376491-1
Atg8a	R	1:2000	WB	Sigma ABC974	3308314
Phosphor-S51 eIF2S	R	1:1000	WB	Cell Signaling 3597	12
eIF2S1	R	1:500	WB	Abcam 26197	GR3183673-1 GR3325905
Puromycin monoclonal	M	1:1000	WB	Kerafast EQ0001	3RH11
Rabbit ECL	G	1:10000	WB	GE healthcare NA931	17473046
Mouse ECL	G	1:10000	WB	GE Healthcare NA934	17170583

Disease Models & Mechanisms • Supplementary information

Table S3. Statistical tests and results from studies.

	Test used/rationale	Genotype comparison	Result (p-value)
Fig 2B	2-tailed Mann-Whitney rank sum test. More conservative non-parametric statistic chosen due to unknown if variance is the same between genotypes due to immunostaining and microscopy methods.	W1118 vs Δ9 homozygote	0.03
Fig 2B		W1118 vs Δ9/Df hemizygote	0.004
Fig 2B		Δ9 homozygote vs Δ9/Df hemizygote	n.s.
Fig 2C		W1118 vs Δ9 homozygote	n.s.
Fig 2C		W1118 vs Δ9/Df hemizygote	0.009
Fig 2C		Δ9 homozygote vs Δ9/Df hemizygote	0.001
Fig 2D		W1118 vs Δ9 homozygote	n.s.
Fig 2D		W1118 vs Δ9/Df hemizygote	n.s.
Fig 2D		Δ9 homozygote vs Δ9/Df hemizygote	n.s.
Fig 2E		W1118 vs Δ9 homozygote	n.s.
Fig 2E		W1118 vs Δ9/Df hemizygote	n.s.
Fig 2E		Δ9 homozygote vs Δ9/Df hemizygote	n.s.
Fig 3C	2-tailed Mann-Whitney rank sum test. More conservative non-parametric statistic chosen due to unknown if variance is the same between genotypes due to immunostaining and microscopy methods.	W1118 vs Δ9 homozygote NMJ	n.s.
Fig 3C		W1118 vs Δ9 homozygote neuron	0.02
Fig 3C'		W1118 vs Δ9 homozygote NMJ	9.596e-05
Fig 3C'		W1118 vs Δ9 homozygote neuron	n.s.
Fig 3C''		W1118 vs Δ9 homozygote NMJ	0.03
Fig 3C''		W1118 vs Δ9 homozygote neuron	0.01
Fig 3F		W1118 vs Δ9 homozygote NMJ	0.003
Fig 3F		W1118 vs Δ9 homozygote neuron	0.02
Fig 3F'		W1118 vs Δ9 homozygote NMJ	n.s.
Fig 3F'		W1118 vs Δ9 homozygote neuron	n.s.
Fig 3F''		W1118 vs Δ9 homozygote NMJ	0.02
Fig 3F''		W1118 vs Δ9 homozygote neuron	0.03
Fig 3G		W1118 vs Δ9 homozygote	0.02
Fig 4B	$p<0.05$ by 2-tailed paired t-test. T-test chosen as biological samples prepared in parallel and normalized to genetic and treatment control.	w^{1118} control vs starve	n.s.
Fig 4B		Control w^{1118} vs *Δ9* homozygote	0.04
Fig 4B		*Δ9* homozygote control vs starve	0.03
Fig 4B		Starve w^{1118} vs *Δ9* homozygote	n.s.
Fig 4C		w^{1118} control vs starve	n.s.
Fig 4C		Control w^{1118} vs *Δ9* homozygote	0.02
Fig 4C		*Δ9* homozygote control vs starve	n.s.
Fig 4C		Starve w^{1118} vs *Δ9* homozygote	n.s.
Fig 4E	$p<0.05$ by 2-tailed paired t-test. T-test	w^{1118} control vs chloroquine	0.009
Fig 4E		Control w^{1118} vs *Δ9* homozygote	0.02

Disease Models & Mechanisms • Supplementary information

Fig 4E	chosen as biological samples prepared in parallel and normalized to genetic and treatment control.	*Δ9* homozygote control vs chloroquine	0.03
Fig 4E		chloroquine w^{1118} vs *Δ9* homozygote	n.s.
Fig 4F		w^{1118} control vs chloroquine	n.s.
Fig 4F		Control w^{1118} vs *Δ9* homozygote	0.05
Fig 4F		*Δ9* homozygote control vs chloroquine	n.s.
Fig 4F		chloroquine w^{1118} vs *Δ9* homozygote	n.s.
Fig 5B	2-tailed paired t-test. T-test chosen as biological samples prepared in parallel and normalized to genetic and treatment control.	w^{1118} vs *Δ9* homozygote 3L	
Fig 5B		w^{1118} vs *Δ9* homozygote 1 day	n.s.
Fig 5B		w^{1118} vs *Δ9* homozygote 14 day	0.003
Fig 5B		w^{1118} 1 day vs 14 days	0.004
Fig 5B		*Δ9* homozygote 1 day vs 14 days	n.s.
Fig 5C		w^{1118} control vs starvation	0.005
Fig 5C		Control w^{1118} vs *Δ9* homozygote	0.06
Fig 5C		*Δ9* homozygote control vs starvation	n.s.
Fig 5C		starvation w^{1118} vs *Δ9* homozygote	n.s.
Fig 5D		w^{1118} 0 v 4 hour tunicamycin	0.03
Fig 5D		w^{1118} 0 v 24 hour tunicamycin	0.03
Fig 5D		w^{1118} 4 v 24 hour tunicamycin	n.s.
Fig 5D		*Δ9* homozygote 0 v 4 hour tunicamycin	n.s.
Fig 5D		*Δ9* homozygote 0 v 24 hour tunicamycin	0.01
Fig 5D		*Δ9* homozygote 4 v 24 hour tunicamycin	n.s.
Fig 5D		w^{1118} v *Δ9* homozygote 0 hour	n.s.
Fig 5D		w^{1118} v *Δ9* homozygote 4 hour	n.s.
Fig 5D		w^{1118} v *Δ9* homozygote 24 hour	n.s.
Fig 6B	2-tailed Mann-Whitney rank sum test chosen because using non-normalized data.	w^{1118} control vs tunicamycin	0.02
Fig 6B		w^{1118} vs *Δ9* homozygote control	0.008
Fig 6B		*Δ9* control vs tunicamycin	n.s.
Fig 6B		w^{1118} vs *Δ9* homozygote tunicamycin	n.s.
Fig 6C	Kaplan meyer log-rank test. This is the standard hypothesis-based, nonparametric test to compare the survival distributions of two samples and accounts for the right-hand skew.	w^{1118} vs *Δ9* homozygote	$p < 2\times10^{-16}$
Fig 6D		w^{1118} vs *Δ9* homozygote	$p < 5\times10^{-16}$

Disease Models & Mechanisms • Supplementary information
